# “Efficacy of Netrin-1 Primed WJMSCs-Derived Secretome in Promoting Wound Healing: An *In vitro* Study”

**DOI:** 10.21203/rs.3.rs-8132161/v1

**Published:** 2025-12-31

**Authors:** Shweta Verma, Jahnavy Madhukar Joshi, Raghavendra Upadhya, Samatha Bhat, Shashikala K Bhat, Raviraja Neelavar Seetharam

**Affiliations:** 1 Department of Biotherapeutics Research, Manipal Academy of Higher Education, Karnataka, Manipal, 576104, India; 2 Department of Obstetrics and Gynaecology, Melaka Manipal Medical College, Karnataka, Manipal, 576104, India; 3 Chandigarh University, NH 95, Chandigarh- Ludhiana Highway, District Mohali, Punjab, 140413

**Keywords:** Netrin-1, Mesenchymal Stromal Cell, Secretome, Priming

## Abstract

Wound healing is a multifaceted biological process that requires tightly regulated cellular activities, including proliferation, migration, and extracellular matrix remodeling. Mesenchymal stromal cells (MSCs) derived from Wharton’s Jelly (WJMSC) have emerged as powerful agents in regenerative medicine, largely attributed to their secretome, a dynamic mixture of bioactive molecules and extracellular vesicles. Recent advancements suggest that priming MSCs can potentiate their secretome’s therapeutic efficacy. However, the ability to selectively enhance wound repair mechanisms via tailored priming remains unexplored.

In this study, we introduce recombinant netrin-1, a multifunctional guidance cue known for its dual role in angiogenesis and immune modulation, as a novel priming agent for WJMSCs. We demonstrate that netrin-1 priming significantly enriches the WJMSCs secretome, amplifying its regenerative potential. Conditioned media collected post 48 hours of netrin-1 PRIMING markedly enhanced fibroblast proliferation and migration, boosted angiogenic tube formation in HUVECs, and skewed macrophage polarization toward an anti-inflammatory M2 phenotype, required as key processes that orchestrates effective tissue repair.

This study clearly establishes for the first time the role of netrin-1 priming in creating a functionally superior WJMSCs secretome that targets multiple facets of wound healing synergistically. Unlike conventional single-factor treatments, this approach leverages the natural versatility of MSC secretome augmented with a well-characterized bioactive cue, offering a sophisticated and cell-free therapeutic avenue. The netrin-1 WJMSC secretome presents a promising platform with translational potential to address complex wound healing challenges, especially chronic and non-healing wounds with persistent inflammation and impaired angiogenesis.

Altogether, the N1-primed WJ-MSC secretome represents a promising, cell-free therapeutic strategy for enhancing wound healing and tissue regeneration. Future studies will focus on the development of targeted delivery systems to optimize their therapeutic efficacy in preclinical wound models, paving the way for advanced regenerative medicine interventions.

## Graphical Abstract

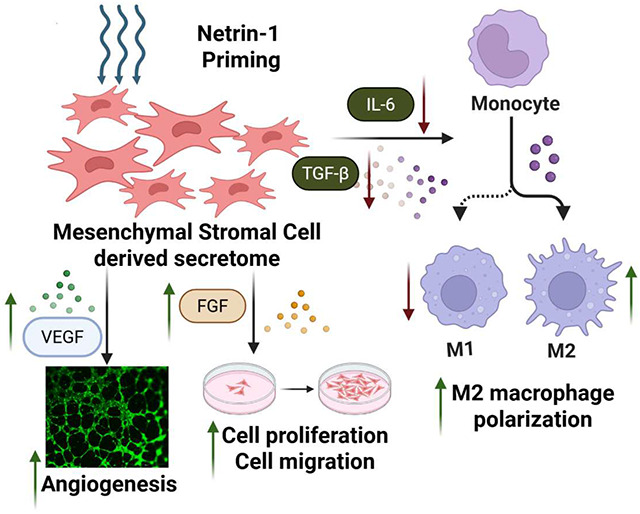

## 1. Introduction

Chronic wounds such as diabetic foot ulcers, deep burns, etc., often fail to heal due to persistent inflammation, impaired vascularization, dysregulated macrophage polarization, and reduced fibroblast activity. These factors contribute to delayed wound closure and compromised tissue regeneration [1,2]. Wharton’s Jelly-derived mesenchymal stem cells (WJMSCs) isolated from gelatinous connective tissue of the umbilical cord have emerged as a promising source for regenerative therapeutics. WJ-MSCs offer advantages over adult-derived MSCs (such as bone marrow or adipose tissue), including non-invasive harvesting, a more primitive stemness, low immunogenicity, and higher proliferative capacity [3,4]. Their fetal-like phenotype allows robust paracrine response, making them appealing for cell-free regenerative applications based on their secretome, which is a complex mixture of proteins, lipids, nucleic acids, and extracellular vesicles (EVs) [5,6]. The WJMSCs-derived secretome plays a crucial role in promoting cell proliferation, angiogenesis, immunomodulation, and extracellular matrix remodeling essential for wound healing.

Key cellular players in wound healing include fibroblasts that produce collagen and structural proteins, endothelial cells that mediate neovascularization, and macrophage cells that coordinate with tissue remodeling and immune resolution. Macrophage plasticity, i.e., M1 to M2 macrophage polarization, is essential for scarless healing [7]. Recent studies have highlighted the potential of priming or preconditioning to enhance the therapeutic potency of MSC-derived secretome. Priming of WJMSCs enhances their paracrine output, enriches the secretome specific to the factor and targeted physiological conditions [8].

Netrin-1, a laminin-related guidance protein originally identified for its role in axon guidance, has gained attention due to its multifunctional role in regeneration in both neuronal and non-neuronal tissues [9]. Netrin-1 interacts with receptors expressed on endothelial cells, macrophages, and fibroblasts, including UNC5B, DCC, and neogenin, to provide proangiogenic, anti-inflammatory, and immunomodulatory effects [10]. By triggering the PI3K/Akt and ERK1/2 signalling pathways, Netrin-1 promotes angiogenic potential and tube formation in endothelial cells [11]. It facilitates the resolution of inflammation by inhibiting monocyte chemotaxis and encouraging a shift toward the M2 anti-inflammatory phenotype in macrophages [12,13]. Additionally, it has been demonstrated that Netrin-1 inhibits tissue fibrosis and regulates matrix deposition and fibroblast migration [14].

Despite the promising multifunctional role of Netrin-1, its therapeutic potential in chronic wound healing remains unexplored. In this study, we hypothesize that stimulation of WJMSCs with Netrin-1 can reprogram the WJMSCs’ secretome to enhance its regenerative and immunomodulatory capacity. This approach may provide a potent cell-free treatment that can alter the proliferation and migration of fibroblasts, angiogenesis mediated by endothelial cells, and macrophage polarization that are essential for faster and effective wound healing.

The objective of this study is to evaluate the *in vitro* functional effects of secretome derived from Netrin-1-primed WJMSCs on three representative cell types involved in wound healing:; i.e. fibroblasts, endothelial cells, and macrophages. This investigation aims to provide mechanistic insights and lay the foundation for future translational applications of primed WJMSCs secretome in chronic wound management.

## 2. Methodology

### 2.1. Ethics statement

The Wharton’s Jelly Mesenchymal stromal cells (WJMSCs) and HUVEC for research purposes was received form healthy volunteer after obtaining written informed consent and approval by the Institutional Ethical Committee, Kasturba Medical College and Kasturba Hospital, Manipal, India (Approval number: IEC 842 2021, and IEC1-220-2025 respectively) and Institutional Committee for stem cell Research (IC-SCR), Manipal Academy of Higher Education (MAHE) (Approval number: MIRM-ICSCR/016/2022).

### 2.2. Isolation of WJMSCs from the Umbilical cord and characterization

WJMSCs were isolated from human umbilical cord obtained from healthy women undergoing Caesarean section, after written informed consent, as previously described [15]. The umbilical cord blood was gently squeezed out, and the umbilical cord was transported in normal saline containing 2 % antibiotic solution (Gibco, Cat# 15240062) at a controlled temperature of 2 – 4 °C. Upon transfer to the GMP laboratory, the umbilical cord was washed three times with normal saline and sectioned into approximately 2 cm pieces. The piece was then longitudinally cut to separate the umbilical vessels, and the Wharton’s jelly was carefully scraped from the cord and minced thoroughly.

A portion of the minced Wharton’s Jelly was transferred into 60 mm culture-treated dishes and incubated with DMEM-LG (Himedia, Cat# AL006), 10 % FBS (Himedia, Cat# RM10679), 1 % L-Glutamine supplement (Himedia, Cat# TCL030), and 0.5 % antibiotic solution (Gibco, Cat# 15240062) at 37 °C in a 5 % CO_2_ incubator. The WJMSCs were allowed to migrate from the tissue explants over seven days, with the culture media changed every other day. Cells exhibiting typical fibroblast-like morphology were observed under the microscope.

The migrated cells at passage zero (P0) were detached by 0.25 % trypsin-EDTA (Himedia, Cat# TCL007) and sub-cultured in a T-75 flask to establish passage one (P1). Upon reaching 85 – 90 % confluency, cells were cryopreserved in freezing media composed of fetal bovine serum (FBS) and dimethyl sulfoxide (DMSO) in a 9:1 ratio, at a density of 0.5 million cells/vial. For characterization and subsequent experiments, WJMSCs were revived and used at passage five (P5).

#### 2.2.1. Cell growth kinetics and population doubling and doubling time assessment

Growth kinetics were determined by seeding cells in triplicate at a seeding density of 5000 cells/cm^2^ in a 6-well plate. Cells were harvested daily up to day *in vitro* 5 (DIV5) and cell counts were recorded using a hemocytometer.

Population doubling (PD) was calculated based on the initial seeding number at DIV0 and the cell count at each subsequent time point using the formula:

$$PD=\left[\text{logN}-\text{logN}0\right]/\text{log}\;2$$

Where N_0_ is the initial cell number plated at DIV0, and N is the average cell number at each subsequent time point.

Doubling time (DT), the time required for the cell population to double, was calculated for each 24 h interval using the formula:

$$\text{Doubling}\;\text{Time}=\left[\left(T\right)*\text{ln}\left(2\right)\right]/\text{ln}\;\left(\frac{N}{N0}\right)$$

Here, N is the number of cells harvested at time T, and N_0_ is the initial number of cells seeded.

### 2.3. Priming of WJMSCs, collection of conditioned media

WJMSCs were seeded at a seeding density of 5000 cells/cm^2^ and cultured in DMEM-Low Glucose medium supplemented with 10 % FBS, 1 % Glutamax, and 0.5 % PenStrep, with media replaced every other day. Upon reaching 70 – 80 % confluency at passage 5, cells were subjected to serum starvation by replacing the medium with serum-deprived DMEM-Knock Out (DMEM-KO) containing 1 % Glutamax and 0.5 % PenStrep for 6 h. Following starvation, the media was substituted with priming medium composed of DMEM-KO supplemented with 1 % Glutamax, 0.5 % PenStrep, 1 % HT supplement, 1 % vitamin, 0.1 % lipid concentrate, and the designated concentration of recombinant human Netrin-1. Cells were incubated under standard culture conditions for 48 h. After 48 h, culture supernatant was collected, centrifuged to remove cellular debris, aliquoted, and stored at −80 °C for downstream analysis.

#### 2.3.1. Characterization of WJMSCs

##### Immunophenotyping by Flow Cytometry (CD34, CD45, CD75, CD105)

WJMSCs were seeded with a density of 5000 cells/cm^2^ at passage 5 in T-25 flasks and incubated at 37 °C in 5 % CO_2._ Media change was done on alternate days. Upon 80–90 % confluency, cells were harvested, and 0.25 million cells/microfuge tube were allocated for immunophenotyping. Cells were washed twice with Dulbecco’s phosphate-buffered saline (DPBS) and resuspended in 100 μL DPBS. Each tube was incubated with the following surface marker antibodies as per the manufacturer’s instructions in each tube as labelled (CD45-FITC, CD34-PE, CD105-PE, and CD73-PE, Elabscience). Cells were incubated for half an hour in the dark, followed by PBS wash. Cell samples were then acquired using a BD Accuri C6 plus flow cytometer and surface marker expression was analysed using FlowJo software (Version 10.9.0).

##### Multilineage differentiation

A multilineage differentiation assay was conducted at passage 5 to assess the ability of WJMSCs to differentiate into adipocytes, osteocytes, and chondrocytes. Differentiation was induced in confluent cultures using specific induction media for each lineage.

Chondrogenic media was composed of DMEM-HG, 10 % FBS, 1 % ITS, 0.5 % ascorbic acid, 0.05 % Dexamethasone, 0.5 % antibiotic solution, and 0.1 % TGF-β1. Adipogenic media was composed of DMEM-HG, 10 % FBS, 0.1 % insulin, 0.1 % BMX, 0.2 % indomethacin, 0.1 % Dexamethasone, 1 % antibiotic. Ostogenic differentiation media was composed of DMEM-HG with 10 % FBS, 1% G-2-P, 0.5 % ascorbic acid, 0.01 % dexamethasone, 1 % glutamax, 1 % antibiotic. Induction media change was done alternate days till DIV21 of differentiation induction. For negative control cultures were maintained in DMEM-HG with 10 % FBS, 1 % glutamax, and 1 % penstrep.

Cell morphology was monitored during each media change. On day twenty-one, lineage-specific staining was performed to confirm differentiation. Adipocytes were stained with Oil Red O (Sigma-Aldrich, Cat#O1391) to observe oil droplets. Alizarin Red (Sigma-Aldrich, Cat# TMS-008-C) staining was done to observe mineral deposition in osteocytes, and Alcian Blue (Sigma-Aldrich, Cat# TMS-101-C) staining was done for glycosaminoglycan detection for chondrocyte differentiation observation. Microscopic images were captured at 10X for adipogenic and osteogenic differentiated cells, and 4X magnified images were captured for chondrocyte differentiated cells.

#### 2.3.2. Apoptosis Assay (Annexin V/PI Staining)

To assess the effect of Netrin-1 priming on WJMSCs cell death, an apoptosis assay was performed using Annexin V and Propidium Iodide (PI) staining. Following conditioned media collection, cells were harvested and washed three times with phosphate-buffered saline (PBS). Cell pellets were resuspended in Annexin V binding buffer. For each sample, 5 μL Annexin V antibody was added and incubated for 30 min in the dark at room temperature. After incubation, the unbound antibody was removed by a single PBS wash, and PI was added immediately prior to acquisition. Stained cell suspensions were acquired using a BD Accuri C6 plus flow cytometer. The data generated was analysed using FlowJo software (Version 10.9.0) to quantify Annexin V^+^/PI^−^ (early apoptotic), Annexin V^+^/PI^+^ (late apoptotic), and Annexin V^−^/PI^−^ (viable) populations.

#### 2.3.3. Preparation of conditioned media and normalization for treatment

Conditioned media collected from WJMSCs cultures were first centrifuged at 2000 × g for 10 min at 4 °C to remove cellular debris. The supernatant was then passed through a 0.22 μm filter to ensure sterility and further clearance of particulate matter. Filtered conditioned media were aliquoted and stored at −20 °C for short-term preservation for functional assays.

For treatment of target cells with WJMSCs secretome, protein concentration in conditioned media was quantified using the BCA protein assay kit (ThermoScientific, Cat# A65453) according to the manufacturer’s instructions. Based on protein quantification, conditioned media were normalized to a concentration of 200 μg/mL/10^6^ cells. This standardized protein dosing was used to treat target cells to reliably assess the functional effects of the secretome across experiments.

### 2.4. Isolation of HUVEC cells from Umbilical Cord and characterization by Flow cytometer (CD31, CD34, CD45, CD73, CD90)

HUVEC were isolated from the human umbilical cord using established protocols [16]. T-25 flasks were coated with 0.5 % gelatin (Himedia, Cat#TCL109) and were air-dried to facilitate cell attachment. Clot-free umbilical cord segments were collected and transported at 2 – 8 °C in sterile normal saline. Cords were thoroughly washed with normal saline to remove residual blood. Both ends of the umbilical cords were trimmed with a scalpel. The umbilical cord vein was flushed with normal saline repeatedly using a sterile syringe. One end of the cord was clamped with a surgical clamp, and 0.5 % dispase (Himedia, Cat# TC-303) was introduced into the vein. The other end was then clamped to prevent leakage, and the cord was incubated at 37 °C for 30 min to facilitate enzymatic digestion.

The enzyme-digested cell suspension was collected into a 50 mL conical tube containing double the volume of media supplemented with FBS to neutralize the enzyme. The resulting solution was centrifuged at 900 × g for 5 min at RT, and the supernatant was discarded. The cell pellet was resuspended in HiEndoXL^™^ Endothelial Cell Expansion Medium with Reduced Serum (HiMedia, Cat #AL530) and cultured in pre-coated T-25 flasks. Cells were maintained at 37 °C in a 5 % CO_2_ humidified incubator, with media changed every other day. Upon reaching confluency, HUVECs were cryopreserved in freezing media and stored in liquid nitrogen for subsequent characterization and functional assays.

#### 2.4.1. Characterization of HUVECs by Flow Cytometry

For immunophenotypic profiling, HUVECs were harvested and stained with fluorochrome-conjugated antibodies against CD31-FITC, CD34-PE, CD45-FITC, CD73-PE, and CD90-FITC (ELabScience). The stained cells were analyzed using a BD Accuri C6 Plus flow cytometer, and the data was analyzed by FlowJo software (Version 10.9.0).

### 2.5. HUVECs functional analysis

#### 2.5.1. Cell Viability Assay / Cell Proliferation Assay

HUVECs were seeded with a seeding density of 5000 cells/well in a 96-well plate using endothelial-specific media. After 24 h of attachment, cells were treated with WJ-MSC-derived secretome (primed and unprimed) or control media consisting of serum-deprived DMEM-KO supplemented with 1 % Glutamax and 0.5 % PenStrep. Following 24 h of treatment, cells were washed with DPBS. Cell viability and proliferation were quantified using WST-8 reagent (Himedia, Cat#CCK074). Briefly, 10 μL of WST-8 reagent was added to each well and incubated at 37 °C, with 5 % CO_2_ for 2 h. Absorbance was measured at 450 nm using a microplate reader. The percentage proliferation was calculated relative to the control wells treated with serum-deprived DMEM-KO media.

%CellProliferation=(ODofTestSample/MeanODofControlSample)*100

#### 2.5.2. Tube formation Assay

To evaluate angiogenic potential, the ability of HUVECs to form capillary-like networks in vitro was assessed using a tube formation assay. Wells of a 96-well plate were coated with 50 μL of Cultrex Reduced Growth Factor Basement Membrane Extract (RnD Systems, #3433-010-01), kept overnight at 4 °C. The coated wells were allowed to solidify at 37 °C with 5 % CO_2_. HUVECs were then seeded at 10,000 cells/ well on the Cultrex matrix and incubated for 6 h at 37 °C, 5 % CO_2,_ in the presence of conditioned media from primed or unprimed WJMSCs. Following incubation, cells were stained with Calcein AM (Invitrogen Cat#C3099) and imaged using EVOS M7000 fluorescent microscope (Thermo Fischer Scientific, USA). Quantitative analysis of the tube formation was performed using the Angiogenesis Analyzer plugin of ImageJ software [17].

### 2.6. Macrophage cells functional analysis

The murine macrophage cell line RAW 264.7 was obtained from the National Centre for Cell Science (NCCS), Pune, India. Cells were cultured in DMEM-HG media supplemented with 10 % FBS, 1 % glutamax, and 0.5 % penstrep. Cultures were maintained at 37 °C in a humidified atmosphere containing 5 % CO_2_ and allowed to reach approximately 80 % confluency. For experiments, cells were seeded in 24-well plates at a density of 30,000 cells/well in 0.5 mL complete media. Upon 80 % confluency, macrophages were stimulated with 100 ng/mL LPS (Sigma-Aldrich, Cat#L4516) for 24 h to induce M1 inflammatory phenotype. Subsequently, the LPS-containing media were replaced with either 1 μg/mL dexamethasone (Sigma-Aldrich, Cat# D1756) as a positive control for anti-inflammatory activation, or WJMSCs-derived secretome for 48 h.

After 48 h of treatment, conditioned media were collected for nitric oxide (NO) quantification. Cells were harvested for subsequent surface marker analysis.

#### 2.6.1. Surface marker analysis

Post treatment, cells were washed with DPBS twice and gently scraped using a cell scraper to detach adherent cells. Cell suspensions were collected in microcentrifuge tubes and washed with DPBS three times to remove residual media. Cells were then incubated with fluorochrome-conjugated antibodies against CD206-APC, CD86-PE antibody (Elabscience), following the manufacturer’s recommended concentration. Staining was performed in the dark at room temperature for 30 min. After incubation, cells were washed with DPBS to remove unbound antibodies, minimizing background fluorescence for flow cytometric analysis. Cells were acquired using a BD Accuri C6 plus flow cytometer and data was analysed by FlowJo software (Version 10.9.0).

#### 2.6.2. Nitric Oxide Quantification by Griess Assay

Conditioned media collected from macrophage cultures were centrifuged at 900 g for 3 min at RT to pellet any residual cells or debris. An equal volume of cleared conditioned media was mixed with Griess reagent (Sigma Aldrich, Cat# G4410) and incubated at room temperature for 15 min. The absorbance of the resulting chromophore was measured at 540 nm using a microplate reader. Nitrite concentrations, as an indicator of nitric oxide production, were quantified by comparison to a standard curve generated with sodium nitrite.

### 2.7. Fibroblast cells (NIH3T3 cell Line) functional analysis

NIH3T3 cells were procured from theNCCS, Pune, India. Cells were maintained in DMEM high glucose (Himedia, Cat# AL-006) with 10 % FBS, 1 % glutamax, and 0.5 % antibiotic solution. Cultures were incubated at 37 °C in a humidified atmosphere containing 5 % CO_2_ with media changed every alternate day. Subculturing was performed before reaching full confluence to maintain exponential growth and avoid contact inhibition.

#### 2.7.1. Cell Proliferation/ Viability assay

NIH3T3 cells were seeded at a density of 5000 cells/well into 96 96-well plate in complete DMEM high glucose. After 24 h, cells were starved for 6 h to synchronize the cell cycle. Subsequently, cells were treated with WJMSCs-derived secretome or control serum-free medium. After 24 h, cells were washed with DPBS, and cell proliferation was assessed by WST-8 assay. Briefly, 10 μL of WST-8 reagent was added to each well and incubated at 37 °C with 5 % CO_2_ for 2 h. Absorbance was measured at 450 nm using a microplate reader. The percentage proliferation was calculated relative to control wells treated with serum-deprived DMEM-KO (Gibco, Cat# 10829-018) medium supplemented with 1 % glutamax and 0.5 % penstrep.

%CellProliferation=(ODofTestSample/MeanODofControlSample)*100

#### 2.7.2. Wound healing assay (Scratch Assay)

The effect of WJMSCs-derived secretome with and without priming on fibroblast migration was evaluated by *in vitro* wound healing assay, as previously described [18]. NIH3T3 cells were seeded at 10,000 cells/well in a 96-well plate containing complete DMEM-HG media and cultured until 85–90 % confluency.

A uniform linear scratch was created using a 10 μL sterile pipette tip. Cells were washed twice gently with DPBS to remove detached cells and debris, then fresh medium containing the secretome treatment was added. Wound areas were imaged immediately and at 6, 12, 24, and 48 h post scratch using an inverted phase contrast microscope.

The percentage of wound closure was quantified using ImageJ software by measuring the scratch area at the initial time and at each time point using the formulae:

$$\%\;\text{Cell}\;\text{migration}=\frac{A0-At}{A0}*100$$

Where A_0_ is the scratch area at t=0 h,

A_t_ is the scratch area at time t (i.e., 24 h, or 48 h)

The area obtained was plotted as % Area covered v/s time. The area was calculated using the ImageJ software.

### 2.8. Statistical analysis

Data is represented as mean ± SD or mean ± SEM. One-way ANOVA analysis was used to analyse the significance between unprimed and primed groups in the study. The difference between the two groups was determined as significant if p < 0.05. All statistical analysis was performed using GraphPad Prism software (version 8.0.2).

## 3. Results

### 3.1. Priming with Netrin-1 did not affect the characteristics of WJMSCs

**Figure 2.**
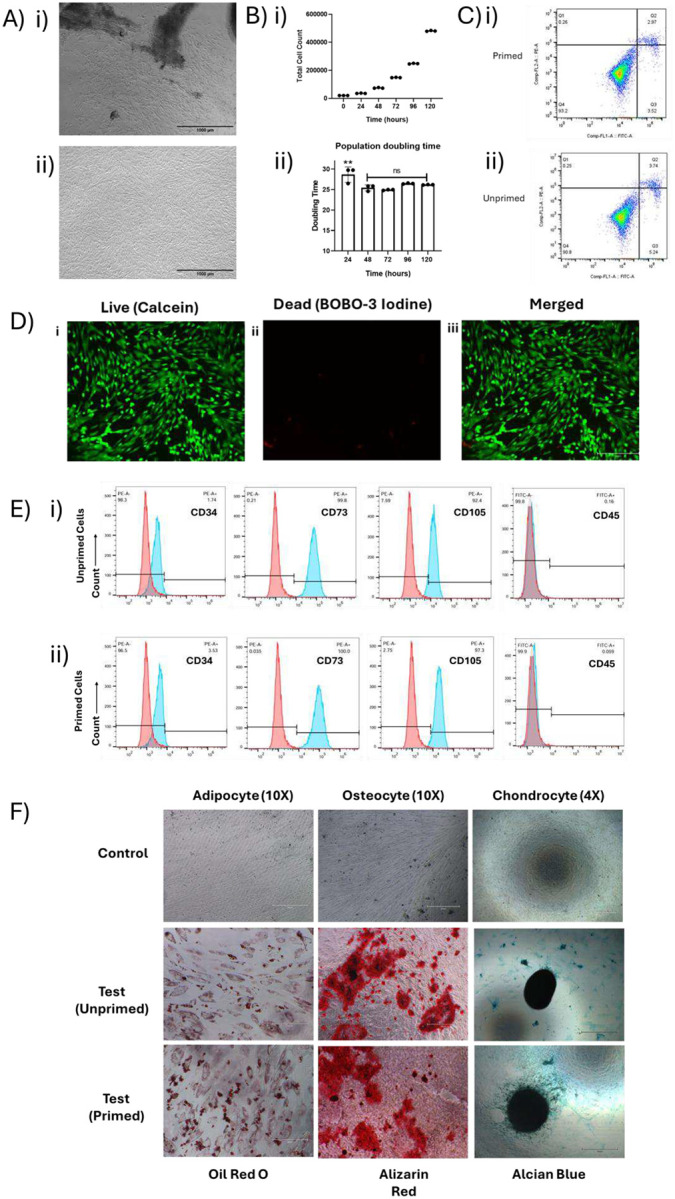
Characterization of unprimed and netrin-1 primed WJMSCs (A) WJMSCs showed fibroblast-like spindle-shaped and (B) population doubling time of 26.31± 0.98 h. The comparative characterization of primed and unprimed MSCs by (C) Flow cytometric analysis showing the expression of negative marker CD34 and CD45 less than 5 % and positive markers CD73 and CD105 more than 95 % in both primed and unprimed WJMSCs (D) Live and dead image of primed WJMSCs with Calcein and BOBO-3 Iodine shows nonsignificant cell death (E) The flow analysis of apoptosis assay of primed and unprimed cells showing less than 10 % apoptotic cells and more than 90 % healthy and live cells (F) Images of cells after tri-lineage differentiation of adipocyte, osteocyte in 10X and chondrocyte in 4X.

The morphology of WJMSCs was found to be fibroblast-like and spindle-shaped (Fig 2a) and remained the same till the 6^th^ passage. The average cell count was consistently increasing from 20,000 cells at DIV0 to approximately 480,667 cells at DIV5, showing sustained proliferation. Population doubling was observed from 0 at DIV0 to 4.59 at DIV5, indicating almost 4.6 population doubling over 5 days. Calculation of doubling time for each 24 hours ranged from 0.95 days to 1.38 days with an overall doubling time of approximately 1.15 days (26.31±0.98 h) (Fig 2b).

WJMSCs cultured with and without netrin-1 exhibited similar surface marker profiles as per the guidelines established by the International Society for Cellular Therapy (ISCT) [19]. Flow cytometric analysis showed that more than 95 % of cells expressed the positive MSC markers CD73 (99.9 % and 100 %) and CD105 (99.6 % and 99.8 %). The expression of hematopoietic markers CD34 (0.31 % and 0.32 %) and CD45 (0.088 % and 0.00 %) was negligible in both unprimed and primed cells respectively (Fig 2e). These results confirm the mesenchymal stem cell identity of WJMSCs under both culture conditions.

Both primed and unprimed WJMSCs demonstrated the capacity to differentiate into adipogenic, osteogenic, and chondrogenic lineages after 21 days of induction. Adipogenic differentiation was confirmed by the accumulation of intracellular lipid droplets visualized with Oil Red O staining. Osteogenic differentiation was validated by mineral deposition, confirmed by Alizarin Red staining. Chondrogenic differentiation was evidenced by the formation of dense cell pellets that stained positively with Alcian Blue stain, indicating proteoglycan-rich extracellular matrix formation (Fig 2f). No significant difference was observed in the differential potential of primed and unprimed cells.

Qualitative analysis of the apoptosis assay showed that priming WJMSCs with Netrin-1 did not significantly affect cell viability. The proportion of early and late apoptotic cells remained below 10 % in both primed and unprimed groups after 48 hours of priming. These findings were corroborated by live/dead imaging, which showed no substantial increase in cell death in the primed conditions compared to controls (Fig. 2d).

### 3.2. Netrin-1 primed WJMSC secretome showed higher angiogenic properties

**Figure 3.**
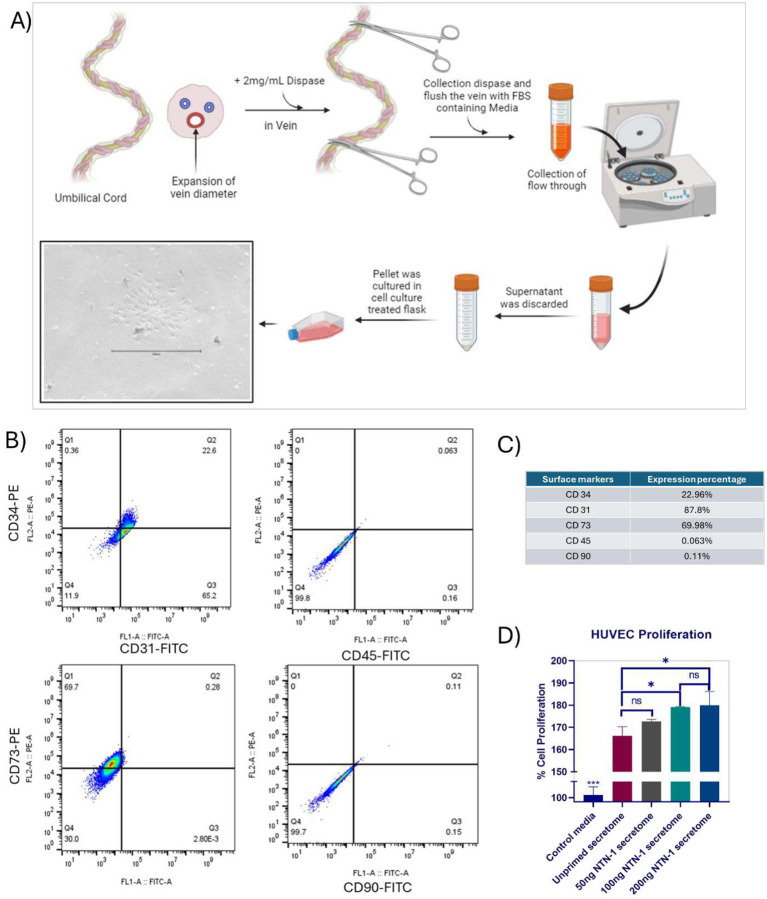
(A) Schematic representation of HUVEC isolation from Umbilical cord, (B) Flow cytometry graph representation of immunophenotypic expression on cells, (C) Expression percentage of surface markers in HUVEC cells (D) Percentage of HUVEC cell proliferation after 24 h of treatment with unprimed secretome in comparison of 50, 100 and 200 ng of Netrin-1 primed MSC secretome (ns=non-significant, *P<0.05; **P<0.01, and ***P<0.001).

Flow cytometric characterization of isolated endothelial cells demonstrated distinct expression of endothelial-specific surface markers. The cells showed 87.8 % positivity for CD31, 22.96 % positivity for CD34, and 69.98 % positivity for CD73, indicating the high presence of endothelial lineage markers. Conversely, negligible expression of hematopoietic and mesenchymal-associated markers CD45 (0.063 %) and CD90 (0.11 %) was observed. These results confirm the successful isolation and identification of endothelial cells (Fig 3b,c).

The proliferation assay demonstrated a significant increase in HUVEC proliferation upon treatment with WJMSCs’ conditioned media compared to the negative control cells. Conditioned media from 50 ng/mL Netrin-1 primed WJMSCs did not show a significant difference in cell proliferation compared to the unprimed secretome. However, treatment with secretome primed at higher concentrations of 100 ng/mL and 200 ng/mL Netrin-1 resulted in significant enhancement in endothelial cell proliferation, with no statistically significant difference observed between the 100 ng/mL and 200 ng/mL treatment groups. (Fig 3d).

**Figure 4.**
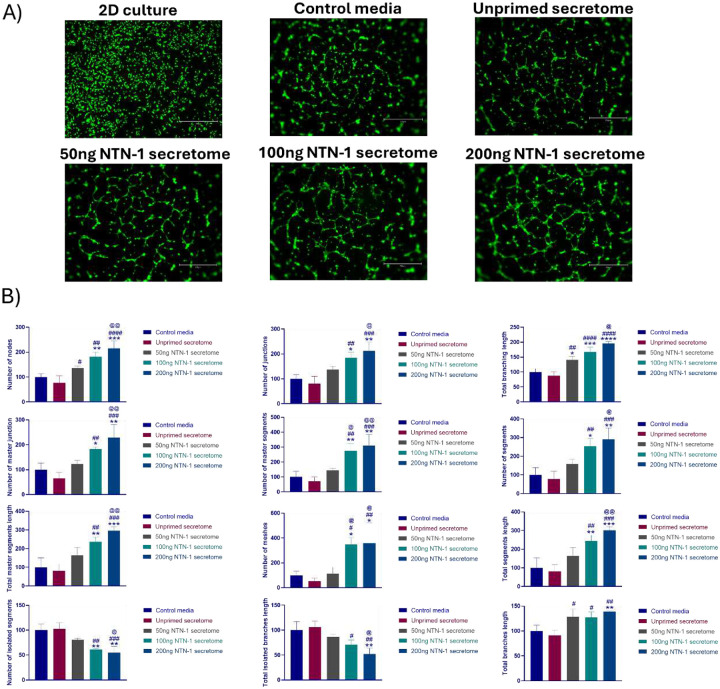
Angiogenic property of WJMSC secretome with and without priming (A) Image representation of tube formation after treatment with primed and unprimed secretome along with 2D culture on surface treated plate without geltrex coating and serum-deprived DMEM-KO control media (B) Graphical representation of analysed images for total tube length, number of extremities, and mean mesh size etc. after treatment analysed by angiogenesis analyzer plugin software by ImageJ (*P<0.05; **P<0.01, ***P<0.001 ****P<0.0001). * denotes the significant difference in comparison with control media (DMEM-KO), # in comparison with unprimed secretome treatment, and @ with 50 ng NTN-1 primed secretome treatment

Consistent with these findings, the tube formation assay revealed that HUVECs treated with secretome primed at 100 ng/mL and 200 ng/mL Netrin-1 formed a significantly greater number of nodes, junctions, segments, mesh areas, and branches, and exhibited an increase of total tube length compared to the negative control, unprimed secretome, and 50 ng/mL primed secretome groups. Similar to proliferation results, no significant differences were found between the 100 ng/mL and 200 ng/mL primed secretome treatments in terms of tube formation metrics (Fig 4a,b).

### 3.3. Netrin-1 primed WJMSC secretome showed enhanced anti-inflammatory effects in macrophage polarization

#### Macrophage Polarization Analysis

**Figure 5.**
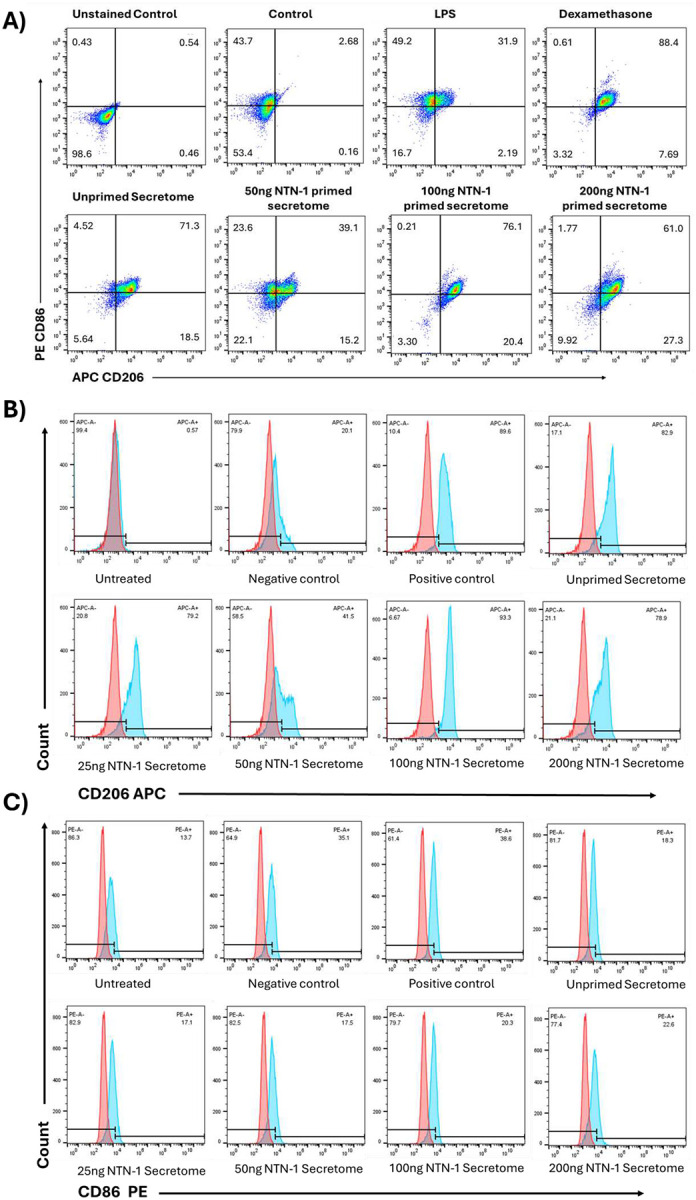
Macrophage polarization ability of netrin-1 primed and unprimed WJMSCs secretome. (A) Flow cytometry representative dot plot of LPS-activated macrophage cells with positive control dexamethasone, and primed and unprimed WJMSCs secretome, (B) Representative flow cytometry histograms of M2 macrophage marker CD206-APC, and (C) Representative flow cytometry histograms of M1 macrophage marker CD86-PE

**Figure 6.**
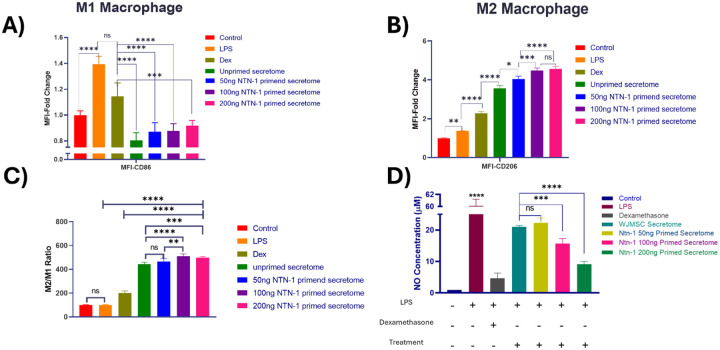
The treatment of Macrophage cells with LPS was done to activate M1 macrophage cells where upon treatment with primed and unprimed secretome reduced the M1 macrophage and enhanced M2 macrophage polarization (A) Graphical representation of M1:M2 after treatments. (B) Median Fluorescence Intensity (MFI) of CD86 that indicated M1 macrophage population (C). Median Fluorescent Intensity of CD206, which indicates the M2 macrophage cell population. The Nitric oxide release and DCFH-DA staining by Macrophage indicates the inflammatory response in the cells respectively (D) Treatment with primed secretome decreases the NO concentration significantly in comparison to unprimed secretome (ns=non-significant, *P<0.05; **P<0.01, ***P<0.001 ****P<0.0001)

Flow cytometric analysis revealed distinct polarization patterns of RAW 264.7 macrophages following different treatments (Fig 5). LPS stimulation increased CD86 expression (M1 marker) by approximately 1.4-fold compared to control, confirming successful M1 polarization induction. Treatment with dexamethasone (positive control) effectively reduced CD86 expression while significantly increasing CD206 expression (M2 marker) by approximately 2.3-fold, demonstrating M2 polarization. WJMSCs-derived secretome treatments showed differential effects on macrophage polarization. Unprimed secretome significantly reduced CD86 expression compared to LPS-treated cells and increased CD206 expression approximately 3.5-fold above control levels. Netrin-1 primed secretome demonstrated enhanced M2 polarization capacity, with CD206 expression reaching 4 to 4.5-fold above control levels. No significant differences were observed between the three netrin-1 concentrations tested. The M2/M1 ratio analysis showed that all secretome treatments significantly increased the M2/M1 ratio compared to LPS treatment, with primed secretome achieving a ratio 4.5-fold more in comparison to LPS alone (Fig 6b, 6c).

Griess assay results demonstrated that LPS stimulation significantly elevated NO production to approximately 60 μM compared to the control. Dexamethasone treatment substantially reduced NO levels to approximately 4.6 μM, consistent with M2 macrophage polarization. WJMSCs secretome treatments significantly reduced NO production compared to LPS treatment, with unprimed secretome reducing levels to approximately 20 μM. Netrin-1 primed secretome at 50, 100, and 200 ng/mL concentrations further reduced NO production to approximately 22, 15.6, and 9 μM, respectively, showing concentration-dependent effect (Fig 6d).

### 3.4. Primed secretome enhances fibroblast functionality in terms of proliferation and migration

**Figure 7.**
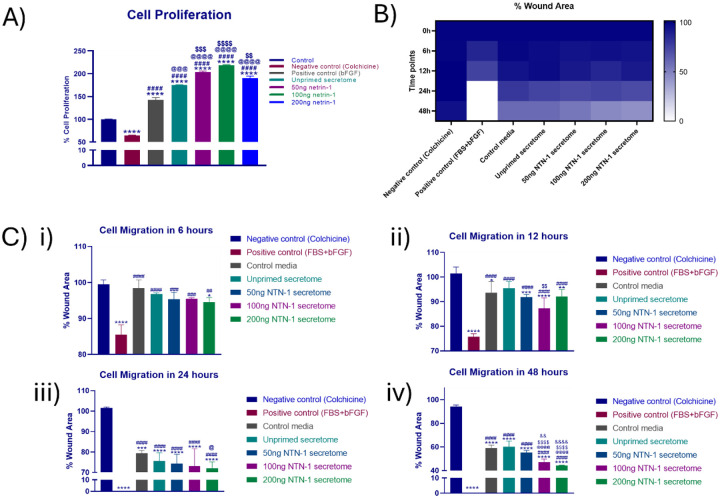
Primed secretome enhances the cell proliferation and migration capabilities of fibroblast cells (A) NIH3T3 cell proliferation after treatment with secretome along with negative control (colchicine), and positive control (bFGF) (B) Representative heatmap of scratch assay to assess cell migration after treatment with secretome, (C) Graphical representation of percentage cell migration after 6, 12, 24, and 48 h (i – iv), * *Denotes the significance with respect to negative control, # with respect to positive control, @ with respect to blank media, $ with respect to unprimed secretome, and & with respect to 50 ng/mL netrin-1 primed secretome (ns=non-significant, *P<0.05; **P<0.01, ***P<0.001 ****P<0.0001)

Cell proliferation assay demonstrated that WJMSCs-derived secretome significantly enhanced NIH3T3 fibroblast proliferation compared to the negative control (colchicine treatment). Unprimed secretome increased cell proliferation to approximately 150 % compared to the control, while the positive control bFGF, known to enhance cell proliferation, reached 140 %. Netrin-1 primed secretome showed concentration-dependent effects with 50ng/mL, 100ng/mL, and 200 ng/mL treatments achieving approximately 180, 220, and 190 % proliferation, respectively. The 100 ng/mL netrin-1 primed secretome demonstrated the highest proliferative effect, significantly superior to both the unprimed secretome and the positive control treatments (Fig 7a).

The heatmap analysis revealed distinct patterns of wound closure over time across different treatments (Fig 7b). At 6 h post scratch, minimal differences were observed between treatments, with wound areas remaining largely intact. By 12 h, secretome-treated groups began showing enhanced closure compared to controls. Quantitative analysis of cell migration showed progressive wound closure over a 48 h observation period. At 6 h, the positive control showed approximately 85 % remaining wound area, while the negative control and unprimed secretome maintained around 95–98 % wound area. Netrin-1 primed secretome at all concentrations showed similar performance to unprimed secretome at the early timepoint (Fig 7c).

By 12 h, differences became more apparent with positive control showing approximately 75 % remaining wound area, while secretome treatments (both primed and unprimed) maintained 90–95 % wound area, indicating slower initial migration compared to FBS + bFGF. At 24 h, significant differences emerged with positive control achieving approximately complete wound closure, unprimed secretome reaching about 75 % closure, and Netrin-1 primed secretome showing concentration-dependent effects. The 100 ng/mL and 200 ng/mL treatments achieved approximately 72 % and 70 % wound closure, respectively.

By 48 h, all secretome treatments demonstrated substantial wound closure. Positive control covered the complete wound area, negative control covered only 5 %, with unprimed secretome covering approximately 40 % of the wound, with 60 % remaining wound area. On the other hand, 50 ng/mL had 55.33 %, 100 ng/mL netrin-1 primed secretome had 47.26 % and 200 ng/mL netrin-1 primed secretome had approximately 44.21 % wound area remaining.

## 4. Discussion

The present study provides a comprehensive assessment of the therapeutic potential of WJMSC secretome after priming with recombinant protein Netrin-1, demonstrating marked enhancement in functional parameters essential for effective wound repair. This study builds on recent progress in WJMSC-based regenerative strategies and a novel modality that addresses the complex pathophysiology of chronic wounds by concurrently modulating fibroblast activity, promoting endothelial function, and regulating immune response.

The WJMSCs isolated exhibit robust proliferative capacity *in vitro*, maintaining exponential growth potential for at least five days under optimised culture conditions. The consistent increase in population doubling values, combined with a short average doubling time of 1.15 days (Fig 2b), reflects rapid and sustained cell division. Importantly, no growth plateau or decline in proliferation rate by day five indicates that confluency and contact inhibition were not limiting factors, underscoring the suitability of these cells for applications requiring rapid expansion, including secretome production for therapeutic use.

Crucially, culturing WJMSCs in serum-deprived or netrin-1-supplemented media did not compromise their characteristic surface marker profile, according to ISCT criteria, as cells retained high CD73 and CD105 expression with negligible levels of hematopoietic markers CD34 and CD45 (Fig 2e). Furthermore, netrin-1 priming preserved the multi-lineage differentiation potential in adipogenic, osteogenic, and chondrogenic lineages, showing that multipotency is maintained even under priming conditions (Fig 2f). The absence of significant apoptosis during priming confirms that netrin-1 is non-cytotoxic, strengthening its candidacy as a safe conditioning agent that enhances secretome functionality without impairing cell viability or phenotype (Fig 2c, 2d).

Also, primary human umbilical vein endothelial cells isolated from umbilical cord tissue were validated by flow cytometry, as reported before in articles by Radermacher et al. and Gruber et al. [20,21]. HUVECs isolated exhibited high expression of the canonical endothelial marker CD31 (87.8 %) and moderate expression of progenitor marker CD34 (22.96 %). The presence of CD73, linked to vascular homeostasis and anti-inflammatory signaling, was substantial (69.98 %), while hematopoietic (CD45) and mesenchymal CD90 contamination remained minimal (0.1 %), confirming the purity of the endothelial population for downstream angiogenesis assays (Fig 3c).

Some studies focusing on cell therapy and WJMSC secretome treatment with netrin-1 have been researched. These studies include netrin-1-transfected BMMSCs or netrin-1 and MSCs co-treatment, which enhance angiogenesis by increasing tube formation and migration of BMMSCs in comparison to the control group for improved ischemic hind limb. Another study by Lee et al. investigated that netrin-1 induces cell proliferation via Inα6β4 coupled with c-Src in hUCB-MSCs [22–24]. To assess the functional effect of secretome collected from netrin-1 primed WJMSC cells, different cells involved in wound healing, such as macrophage cells for inflammation, HUVEC for angiogenic properties, and fibroblast cell line for effect in proliferation and migration, were evaluated after treatment with secretome.

Treatment of HUVECs with WJMSCs secretome demonstrated similar results to the literature and showed enhanced tube formation in comparison to treatment without secretome. Comparison of the WJMSC secretome with the primed secretome showed enhancement in angiogenic properties directly proportional to the concentration of Netrin-1 used for priming of MSCs. The WJMSC primed with Netrin-1 at 100 and 200 ng/mL induced significant proliferation compared to the unprimed secretome and control groups, indicating effective enhancement of endothelial growth and angiogenic properties (Fig 4a). The comparable effects at these concentrations suggest a saturation threshold in priming response. The study by Prieto et al. evaluated the effect of netrin-1 secreted by WJMSCs on the endothelial cells that also showed tube formation at 100 ng/mL treatment [25]. Correspondingly, tube formation assays showed improved network complexity, affirming the proangiogenic influence of the primed secretome. Enhanced formation of nodes, junctions, and overall network complexity reflects improved endothelial organization essential for neovascularization. This is mechanistically supported by studies demonstrating that netrin-1 acts directly on endothelial cells through receptors UNC5B and DCC to stimulate PI3K/Akt and ERK1/2 signaling pathways crucial for angiogenesis [26]. Moreover, the primed secretome likely contains enriched extracellular vesicles carrying angiogenic cargo including matrix metalloproteinases, VEGF, and von Willebrand factor, which collectively enhance endothelial function [26,27].

In terms of inflammation, the netrin-1 primed secretome robustly directed macrophages toward that M2 anti-inflammatory phenotype, evidenced by increasing CD206 and decreased CD86 expression alongside reduced nitric oxide production (Fig 6d). This shift from proinflammatory M1 to reparative M2 macrophages is pivotal in resolving chronic inflammation and promoting wound repair through the secretion of cytokines such as IL-10 and TGF-β, and facilitating tissue remodeling and angiogenesis through the production of growth factors and matrix remodeling enzymes [29,30].

Netrin-1 regulates COX-2 expression at the transcriptional level by regulating the activation of NF-κB and promotes macrophage polarization towards M2 phenotype that is anti-inflammatory in nature and supports tissue repair and angiogenesis [31,32]. Studies till now related to netrin-1 in combination with MSCs or MSC secreted netrin-1 have focused on angiogenesis. In this study, along with angiogenesis, we have evaluated the effect of netrin-1 preconditioned WJMSC secretome therapeutic efficacy on fibroblast and macrophage cells, as well to evaluate the effect on wound healing.

The observed concentration-dependent NO reduction suggests that a higher Netrin-1 priming dose more effectively suppresses inflammatory responses, highlighting distinct bioactivity requirements for immune modulation relative to endothelial regeneration. This mechanism aligns with the literature demonstrating that MSC secretome can effectively modulate macrophage polarization through paracrine signaling involving cytokines, growth factors, and extracellular vesicles [33,34]. The optimal effect observed at 200 ng/mL Netrin-1 indicates distinct bioactivity requirements for immune modulation relative to endothelial regeneration (Fig 4).

Fibroblast cells treated with netrin-1 primed secretome, showed enhanced proliferation compared to unprimed secretome and positive controls, with an optimal effect at 100 ng/mL (Fig 7a). This enhancement is particularly significant given that dermal fibroblast migration and proliferation are key discriminators of effective wound healing. The secretome likely supplies a synergistic mixture of growth factors, including Transforming Growth Factor-β (TGF-β), Vascular Endothelial Growth Factor (VEGF), Platelet Derived Growth Factor (PDGF), and Interleukin-1β (IL-1β), anti-inflammatory mediators, and extracellular matrix remodelling proteins that collectively drive fibroblast [29,35].

The concentration-dependent response with optimal effects at 100 ng/mL suggests a specific threshold for maximal growth factor secretion. The secretome likely supplies a synergistic mixture of growth factors, anti-inflammatory mediators, and extracellular matrix remodelling proteins that collectively drive fibroblast expansion and functionality. Similar study by Prieto et al. showed that netrin-1 secreted by WJMSCs showed optimal result in endothelial cell migration, angiogenesis and Chicken chorioallantoic membrane (CAM) assay which is in line with our observations [24].

The analysis reveals that in the initial 6–12 h period, minimal migration was there, representing the lag phase where cells respond to injury and begin mobilizing repair mechanisms. Enhanced closure was observed from 24 to 48 h in primed secretome-treated groups (Fig 7). The primed secretome elicited superior wound closure, notably at higher netrin-1 concentrations, compared to the unprimed secretome. These findings suggest that the netrin-1 priming creates a more potent and comprehensive wound healing secretome by enhancing the regenerative capacity of WJMSCs, making it suitable for treating complex wounds that require both cellular proliferation and directed migration for effective healing.

In summary, our study confirms that Netrin-1 priming significantly enhances the functionality of WJMSC secretome in comparison to unprimed secretome. This finding opens a new possibility for dissecting the complex molecular and cellular mechanisms underlying chronic wound healing or regeneration. Our study highlights that the secretome can be modified to produce an effective cell-free therapeutic product.

Future research should focus on a thorough understanding of molecular profiling of Netrin-1 primed secretome, identification of signature factors involved to enhance chronic wound healing and evaluation of delivery strategies that ensure targeted and sustained action at the wound area. Additionally, for translational study *in vivo* validation, dose optimization and translational investigations are critical to completely disclose the therapeutic potential for chronic wounds and other regenerative indications. These efforts can pave the way for the development of robust, standardized and effective secretome-based products to improve wound care and improve patient outcomes.

## 5. Conclusion

Chronic and non-healing wounds remain a major clinical challenge due to persistent inflammation, impaired angiogenesis, and defective tissue regeneration. Although cell-based therapies involving MSCs have been encouraging, heterogeneity in therapeutic responses and issues with cell transplantation constrain their general clinical application. MSC secretome, which is full of bioactive factors and EVs, presents an attractive cell-free alternative, yet enhancing its regenerative potential is a pressing research imperative.

This study shows that the regenerative potential of WJMSCs-derived secretome is greatly increased upon priming with recombinant netrin-1. To address the fundamental cellular mechanisms underlying tissue repair, the netrin-1 primed secretome effectively stimulates important wound healing processes such as fibroblast migration and proliferation, endothelial angiogenesis, and macrophage polarization toward an anti-inflammatory phenotype. Crucially, the primed secretome accomplishes these goals without compromising cell viability or causing cytotoxicity with saturation of functional activity at 100ng/mL priming.

These findings highlight the uniqueness of utilizing non-canonical guidance molecules to enhance MSC paracrine activity, presenting a multimodal strategy that addresses primary cellular participants necessary for wound repair. The study presents a scalable, cell-free regenerative therapy that surpasses conventional secretome function by enhancing immunomodulation and vascular regeneration synergistically. Future work will focus on optimization of the delivery mechanism and validating preclinical wound models, aiming to accelerate the transition of secretome-based therapy into safe and effective treatments. This study provides a basis for next-generation regenerative therapies that can substantially improve wound healing outcomes in patients with chronic cutaneous injury.

## Supplementary Material

Supplementary Files

This is a list of supplementary files associated with this preprint. Click to download.

- GraphicalAbstract.png

## Figures and Tables

**Figure 1.**
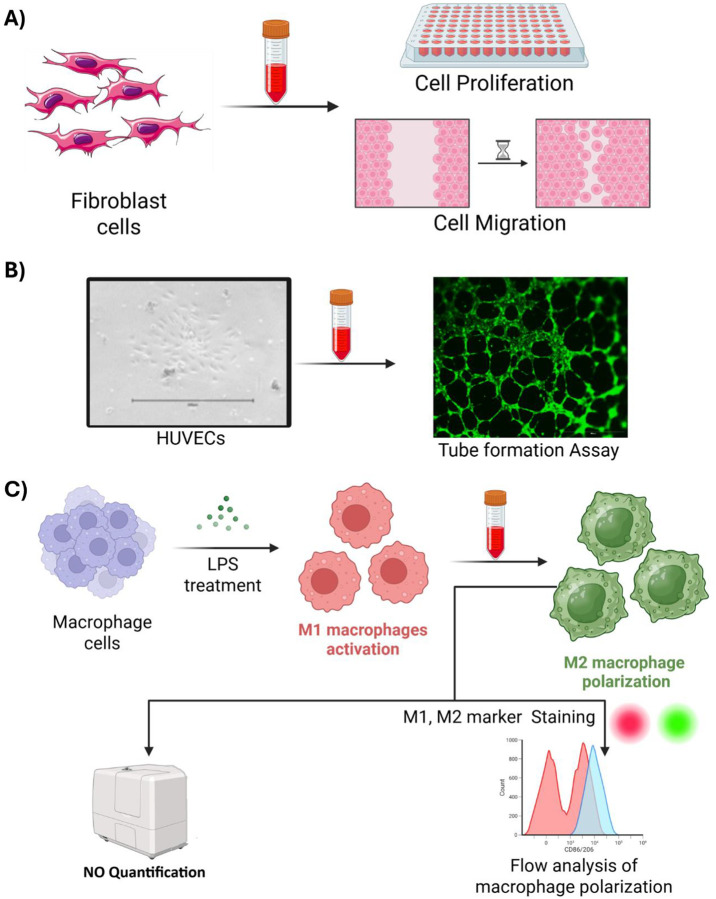
The figure summarize the functional assays performed in the article with different cell types involved in chronic wound healing (A) Fibroblast cell proliferation and migration was performed after treatment with primed and unprimed WJMSC secretome (B) HUVEC cells were treated with secretome to evaluate its tube formation capacity (C) Macrophage cell lines were activated by LPS treatment for M1 macrophage phenotype followed by treatment with the secretome to evaluate the polarization into anti-inflammatory M2 macrophage phenotype. The polarization and functionality was evaluated by flow cytometry and nitric oxide quantification.
